# Identifying Longer-Term Health Events and Outcomes and Health Service Use of Low Birthweight CALD Infants in Australia

**DOI:** 10.1007/s10995-023-03819-w

**Published:** 2023-11-18

**Authors:** Shae Karger, Emmanuel U. Ndayisaba, Joanne Enticott, Emily J. Callander

**Affiliations:** 1 Faculty of Health, University of Technology Sydney, Sydney, Australia; 2 Monash Health, Monash University, Melbourne, Australia; 3 Monash Centre for Health Research and Implementation, Monash University, Melbourne, VIC Australia; 4 School of Public Health and Preventive Medicine, Monash University, 553 St Kilda Rd, Melbourne, VIC 3004 Australia

**Keywords:** Birthweight, Birth outcomes, CALD, Culturally and linguistically diverse, Low birthweight, Marginalised, Pregnancy

## Abstract

**Introduction:**

Approximately one-third of all births in Australia each year are by culturally and linguistically diverse (CALD) women. CALD women are at an increased risk of adverse pregnancy and birth outcomes including prematurity and low birthweight. Infants born weighing less than 2500 g are susceptible to increased risk of ill health and morbidities such as cognitive defects including cerebral palsy, and neuro-motor functioning.

**Methods:**

An existing linked administrative dataset, Maternity 1000 was utilized for this study which has identified all children born in Queensland (QLD), Australia, between 1st July 2012 to 30th June 2018 from the QLD Perinatal Data Collection. This has then been linked to the QLD Hospital Admitted Patient Data Collection, QLD Hospital Non-Admitted Patient Data Collection, QLD Emergency Department Data Collection, and Medicare Benefits Schedule and Pharmaceutical Benefits Scheme Claims Records between 1 and 2012 to 30th June 2019.

**Results:**

Culturally and linguistically diverse infants born with low birthweight had higher mean and standard deviation of all health events and outcomes; potentially preventable hospitalisations, hospital re-admissions, ED presentations without admissions, and development of chronic diseases compared to non-CALD infants born with low birthweight.

**Discussion:**

Results from this study highlight the disparities in health service use and health events and outcomes associated with low birthweight infants, between both CALD and Australian born women. This study has responded to the knowledge gap of low birthweight on the Australian economy by identifying that there are significant inequalities in access to health services for CALD women in Australia, as well as increased health events and poor birth outcomes for these infants when compared to those of mothers born in Australia.

**Supplementary Information:**

The online version contains supplementary material available at 10.1007/s10995-023-03819-w.

## Significance

*What is already known on this subject?* It is known that barriers to care during pregnancy contribute to increased risk of poor birth outcomes such a prematurity, low birthweight, and stillbirth. It is also known that the effects of low birthweight can include costly morbidities, that can extend well into adulthood.

*What this study adds?* First and foremost, this study adds details of poor birth outcomes in Australia for Culturally and Linguistically Diverse women, which previously had limited literature.

**Supplementary Information:**

The online version contains supplementary material available at 10.1007/s10995-023-03819-w.

## Introduction

In Australia, Culturally and Linguistically Diverse (CALD) infants make up one-third of Australia’s births every year (n = ~ 80,000) (Karger et al., 2022; Yelland et al., 2019). CALD women in Australia are at an increased risk of adverse pregnancy and birth outcomes compared to other women, this includes increased risk of low birthweight (LBW) prematurity, infant mortality and stillbirth (Rogers et al., 2020). It is believed that the barriers that may affect the ability of CALD women to access appropriate care are influencing some of these poor birth outcomes. These barriers to care during pregnancy include cultural differences, language barriers, limited health literacy, insufficient support, transport issues and limited financial capacity (Rogers et al., 2020).

Additionally, low birthweight infants that require additional services after discharge often have hospital readmissions, increased contacts with general practitioner’s, educational needs, additional care requirements, and travel costs incurred for parents to have care provided by a healthcare professional (Petrou et al., 2019).

It is speculated that low birth weight may be associated with child health and behaviour problems (Taylor et al., 2001). Infants who are born with LBW (under 2500 g) can be at an increased risk of ill health and morbidities such as including cerebral palsy, problems with cognition, attention and neuro-motor functioning (Hack et al., 1995). Although the risk of these morbidities decreases as the infants weight increases, the effects of such substantial cognitive effects have been are still apparent in adolescent for those who were born with LBW (Hack et al., 1995). Long-term consequences of LBW can also include chronic kidney disease, stroke, cardiomyopathy, and diabetes (Reyes & Manalich, 2005).

**Fig. 1:**
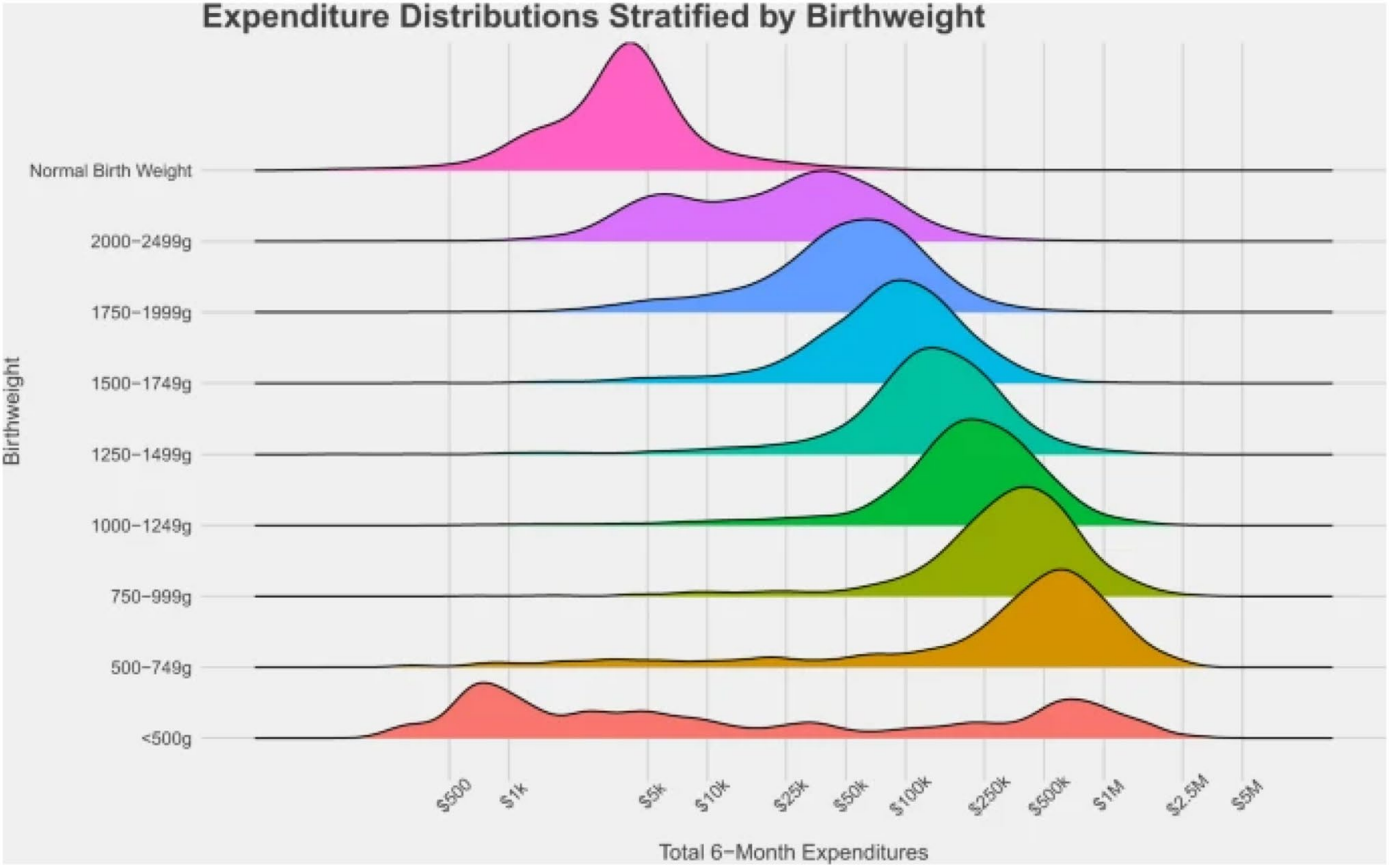
6-month spending distributions, stratified by birthweight, for commercially insured infants 2008–2016 (Beam et al., 2020)

The life-long and long-term health consequences of what happens during birth has been widely studied, identifying several long-term health implications associated with poor birth outcomes not only during early years and adolescence, but also as an adult. Morbidities associated with low birthweight can include problems with cognition, attention and neuro-motor functioning, and these consequences can remain throughout adolescents and into adulthood (Hack et al., 1995). There is also additional literature that confirms that delayed cognitive development is related to poor school performance and behaviour problems (Larson, 2007) (Fig. 1).

Thus, the purpose of this paper was to identify the difference in health outcomes and health service use between CALD infants born with low birthweight compared to CALD infants born at regular birthweight.

## Methods

The research aims guiding this paper were 1: to identify the population-level health service utilization and health events and outcomes of the heterogeneous group of CALD infants and children born in Queensland, Australia, who were born with low birthweight between 1 and 2012 to 30th June 2019, in comparison to CALD infants born at regular birthweight, 2: to identify the costs associated with CALD infants accessing health services between birth and 5 years of age for CALD children born with low birthweight compared to CALD children born with birthweight above 2500 g, and 3: to compare low birthweight inequalities and outcomes in early childhood between infants of CALD women and infants of Australian-born women.

### Culturally and Linguistically Diverse

As defined by the Australian Bureau of Statistics, CALD is defined as individuals born in non-main-English speaking countries. These main-English speaking countries include Australia, Canada, Republic of Ireland, New Zealand, South Africa, United Kingdom (England, Scotland, Wales, Northern Ireland) and United States of America (Australian Bureau of Statistics, 2016). CALD infants were defined by assessing the country of birth of women in the dataset, that had been prearranged into 30 countries or continental regions. Women who were not born in the main-English speaking countries described above, were included in the separate variable for CALD women. These countries and regions included Caribbean, central America, central Asia, central and west Africa, Chinese Asia, eastern Europe, Japan and the Koreas, mainland south-east Asia, maritime south-east Asia, Melanesia, Micronesia, middle east, north Africa, northern Europe, Polynesia, south America, south-eastern Europe, southern Asia, southern Europe, southern and east Africa, southern and eastern Europe, western Europe.

Separate populations were also created for south-east Asian mother’s, European women, African women, and all Asian women (excluding those previously identified as south-east Asian women). These were created in order to compare the results between varying CALD populations. South-east Asian women were defined as those women born in Melanesia, Micronesia, mainland south-east Asia, or maritime south-east Asia. The variable created for women born in Africa was comprised of women born in central and west Africa, north Africa, south and east Africa. South Africa was previously identified by the ABS as a main-English speaking country, and therefore would ideally be excluded from the initial CALD variable and the African mother’s variable that were created. However, for this particular dataset the country of birth data for both southern Africa and east Africa had to be grouped together. Therefore, south Africa was included as a non-main English-speaking country. The variable for women born in Europe was created by including women born in eastern Europe, northern Europe, south-eastern Europe, southern Europe, southern and eastern Europe, and western Europe. The variable for all Asian women (except those previously identified as south-east Asian women) included women born in southern Asia, Japan and the Koreas, Chinese Asia (including Mongolia) and central Asia.

### Dataset

An existing linked administrative dataset, Maternity1000 was utilized for this component of the study. This dataset has identified all infants born in QLD, between 1st July 2012 and 30th June 2018 from the Queensland (QLD) Perinatal Data Collection. This has then been linked to the QLD Hospital Admitted Patient Data Collection, QLD Hospital Non-Admitted Patient Data Collection, QLD Emergency Department Data Collection, and Medicare Benefits Schedule and Pharmaceutical Benefits Scheme Claims Records between 1 and 2012 to 30th June 2019. We included infants born from singleton pregnancies only.

### Analysis Methods

CALD infants were identified on the Maternity 1000 dataset, and a separate population was created. Health events and outcomes for the infants were classified into the following:

Birth outcomes:

- Special care nursery and/or NICU admission at birth.
- APGAR score < 4 at 5 min.
- Stillbirth or neonatal death.
- Prematurity.
- Special care nursery and/or NICU admission at birth or within 30 days of birth.

Health access of low quality:

- Potentially preventable hospitalisations: hospital admissions that could have been prevented by timely and adequate health care in the community (Australian Government, 2019) This was defined as any admission to hospital that could have been prevented by early health intervention through primary care and allied health services.
- Emergency department presentations without admissions,

Onset of chronic health conditions before the age of 5:

- Any hospital admission for infections, cancer, metabolic, mental health, gastrointestinal, neurological, musculoskeletal, cardiovascular, or renal as classified by ICD-10 code assigned to inpatient episodes as principal diagnosis; presentation for mental health outpatient care, based on diagnosis; access to autism, pervasive development disorder and disability service MBS items (Item Numbers 135, 137, 139, 82,015, 82,020, 82,025, 82,035).

Health service use were classified into the following groups:

- GP Consultation.
- Specialist consultations.
- Diagnostic tests and imaging.
- Pathology tests.
- ED presentations.
- In-patient episodes; and.
- Hospital outpatient episodes.

Health service use per year were presented from birth to 5 years.

Initial descriptive statistics were utilized to characterize the difference in demographic and clinical characteristics of the women of CALD infants. To identify the differences in the likelihood of health events and outcomes, we created a series of multivariable logistic regression models to calculate the adjusted odds ratios for each health event or outcome from CALD infants born with low birthweight, compared to those born with regular birthweight. The difference in the number of health services accessed were analysed with Poisson regression models. The difference in number of services accessed were compared for CALD infants born low birthweight, compared to those born with regular birthweight. A negative binomial distribution with a log link function was selected to account for the skewed distribution of the data. All models were adjusted for mother’s age, smoking status at 20 weeks, parity, rurality, socioeconomic status, and calendar year. As a secondary analysis, the data was separated into groups based on CALD geographical location. These groups include south-east Asian (SEA), European, African, Central and South American, and other Asian (excluding SEA) women. Analysis for health service use and health events and outcomeswere also calculated for those infants of Australian-born women in the specified time period.

## Results

Of the n = 326,638 women that gave birth in Queensland, Australia between 1st July 2012 and 30th June 2018, 263,599 were Australian born, and 63,039 were culturally and linguistically diverse. There were 6.55% low birthweight infants from CALD women and 6.17% low birthweight infants from non-CALD women. Table 1 shows that for women aged 21–34 and 35+, CALD women had higher proportions of low birthweight infants compared to Australian-born women (6.17% and 5.80% vs. 7.79% and 7.20% respectively). Demographic characteristics from the majority of women included in this study showed they had previous pregnancies, were married/ de facto, did not smoke during pregnancy, and lived in a major city.

**Table 1:** Characteristics of women who gave birth to LBW and non-LBW CALD children from 1st July 2012 to 30th June 2018

Characteristics	Australian-born women			CALD women		
	n	LBW	Not LBW	n	LBW	Not LBW
Entire sample (326,638)	263,599 (80.70%)	16,265 (6.17%)	247,334 (93.83%)	63,039 (19.30%)	4131 (6.55%)	58,908 (93.45%)
Mother’s age at birth						
< 20 (16,639)	15,789 (94.90%)	1,317 (8.34%)	14,472 (91.66%)	850 (5.10%)	65 (7.65%)	785 (92.35%)
21–34 (254,523)	206,580 (81.16%)	11,980 (5.80%)	194,600 (94.20%)	47,943 (18.84%)	2,956 (6.17%)	44,987 (93.83%)
35+ (55,476)	41,230 (74.32%)	2,968 (7.20%)	38,262 (92.80%)	14,246 (25.68%)	1,110 (7.79%)	13,136 (92.21%)
Mother’s pre-pregnancy BMI,	16,265			4131		
Underweight (< 18.5) (62,736)	17,139 (27.32%)	2,545 (14.85%)	14,594 (85.15%)	45,597 (72.68%)	610 (10.11%)	44,987 (89.89%)
Normal (18.5–24.9) (164,867)	127,345 (77.24%)	7,621 (5.98%)	119,724 (94.02%)	37,522 (22.76%)	2,258 (6.02%)	35,264 (93.98%)
Pre-obesity (25.0–29.9) (72,944)	60,527 (82.97%)	3,056 (5.05%)	57,471 (94.95%)	12,417 (17.03%)	817 (6.58%)	11,600 (93.42%)
Obesity class I (30–34.9) (36,023)	31,736 (88.09%)	1,585 (4.99%)	30,151 (95.01%)	4,287 (11.91%)	289 (6.74%)	3,998 (93.26%)
Obesity class II (30–39.9+) (3,953)	3,555 (89.93%)	176 (4.95%)	3,379 (95.05%)	398 (10.07%)	24 (6.03%)	374 (93.97%)
Area-based socioeconomic status at birth						
Quintile 1 (Lowest), N (%) (20,803)	19,449 (93.49%)	1,414 (7.27%)	18,035 (92.73%)	1,304 (6.51%)	91 (6.98%)	1,213 (93.02%)
Quintile 2, N (%) (11,346)	10,229 (90.15%)	731 (7.15%)	9,498 (92.85%)	1,117 (9.85%)	77 (6.89%)	1,040 (93.11%)
Quintile 3, N (%) (65,882)	52,913 (80.31%)	3,420 (6.46%)	49,493 (93.54%)	12,969 (19.69%)	835 (6.44%)	12,134 (93.56%)
Quintile 4, N (%) (137,153)	118,752 (86.58%)	7,005 (5.90%)	111,747 (94.10%)	18,401 (13.42%)	1,187 (6.45%)	17,214 (93.55%)
Quintile 5 (Highest), N (%) (87,870)	59,021 (67.17%)	3,327 (5.64%)	55,694 (94.36%)	28,849 (32.83%)	1,878 (6.51%)	26,971 (93.49%)
Rurality of residence at birth						
Major city, N (%) (161,425)	116,054 (71.90%)	6,766 (5.83%)	109,288 (94.17%)	45,371 (21.10%)	2,924 (6.44%)	42,447 (93.56%)
Inner regional, N (%) (61,493)	54,519 (88.66%)	3,452 (6.33%)	51,067 (93.67%)	6,974 (11.64%)	453 (6.50%)	6,521 (93.50%)
Outer regional, N (%) (67,211)	59,498 (88.52%)	3,775 (6.34%)	55,723 (93.66%)	7,713 (11.48%)	508 (6.59%)	7,205 (93.41%)
Remote and very remote, N (%) (32,875)	30,293 (92.15%)	1,904 (6.29%)	28,389 (93.71%)	2,582 (7.85%)	183 (7.09%)	2,399 (92.91%)
Mother’s Tobacco smoking status at 20 weeks’ gestation						
Yes, N (%) (38,966)	37,547 (96.36%)	4,381 (11.67%)	33,166 (88.33%)	1,419 (3.64%)	115 (8.10%)	1,304 (91.90%)
No, N (%) (286,704)	225,249 (78.57%)	11,690 (5.19%)	213,559 (94.81%)	61,455 (21.43%)	3,970 (6.46%)	57,485 (93.54%)
Marital status						
Divorced (1,342)	1,029 (76.68%)	98 (9.52%)	931 (90.48%)	313 (23.32%)	26 (8.31%)	287 (91.69%)
Married (Registered) (41,779)	31,411 (75.18%)	1,690 (5.38%)	29,721 (94.62%)	10,368 (24.82%)	682 (6.58%)	9,686 (93.42%)
Married/De Facto (232,376)	184,612 (79.45%)	10,170 (5.51%)	174,442 (94.49%)	47,764 (20.55%)	3,038 (6.36%)	44,726 (93.64%)
Never married (47,380)	43,433 (91.67%)	4,030 (9.28%)	39,403 (90.72%)	3,947 (8.33%)	321 (8.13%)	3,626 (91.87%)
Not stated/unknown (243)	156 (64.20%)	22 (14.10%)	134 (85.90%)	87 (35.80%)	12 (13.79%)	75 (86.21%)
Separated (3,329)	2,823 (84.80%)	245 (8.68%)	2,578 (91.32%)	506 (15.20%)	47 (9.29%)	459 (90.71%)
Widowed (189)	135 (71.43%)	10 (7.41%)	125 (92.59%)	54 (28.57%)	5 (9.26%)	49 (90.74%)
PREV Preg						
Yes (225,716)	184,541 (81.76%)	11,000 (5.96%)	173,541 (94.04%)	41,175 (18.24%)	2,425 (5.89%)	38,750 (94.11%)
No (100,918)	79,054 (78.33%)	5,264 (6.66%)	73,790 (93.34%)	21,864 (21.67%)	1,706 (7.80%)	20,158 (92.20%)
Gest 1st ANC visit						
< 20 weeks (303,636)	247,334 (81.46%)	14,187 (5.96%)	223,711 (94.04%)	56,302 (18.54%)	3,692 (6.56%)	52,610 (93.44%)
20 + weeks (23,002)	16,265 (70.71%)	2,078 (8.09%)	23,623 (91.91%)	6,737 (29.29%)	439 (6.52%)	6,298 (93.48%)

Overall, there were 326,638 infants born from singleton pregnancies (n = 326,638 women)

Table 2a shows the health events and outcomes for low birthweight CALD infants, compared to non-low birthweight CALD infants. Table 2b shows health events and outcomes for low birthweight CALD infants compared to low birthweight non-CALD infants (infants born to Australian born women). CALD infants born with low birthweight had higher mean and standard deviation of all health events and outcomes, potentially preventable hospitalisations, hospital re-admissions, ED presentations without admissions, and development of chronic diseases compared to non-CALD infants born with low birthweight. CALD infants born with low birthweight are 57.68 times more likely to also be born prematurely.

**Table 2:** Health events and outcomes of children born 1st July 2012 to 30th June 2018

(a) Characteristics	LBW CALD children n(%)	Non-LBW CALD children n(%)	aOR	95% Confidence interval		P Value
				Lower	Upper	
Potentially preventable hospitalisations	377 (9.13%)	4,476 (7.6%)	0.79	0.71	0.89	< 0.001
Hospital re-admission(s)	2,759 (66.79%)	7,552 (12.82%)	0.07	0.07	0.08	< 0.001
Emergency department presentations without admission(s)	1,178 (28.52%)	14,824 (25.16%)	0.82	0.77	0.89	< 0.0001
Development of chronic health conditions	571 (14.75%)	5,517 (9.38%)	0.58	0.53	0.64	< 0.0001
Stillbirth	255 (6.17%)	62 (0.11%)	0.02	0.01	0.02	< 0.0001
Prematurity	2,655 (59.4%)	1,476 (2.25%)	57.68	53.10	62.66	< 0.0001
APGAR < 4	406 (9.83%)	245 (0.42%)	0.04	0.03	0.05	< 0.0001

(b) LBW	Non-CALD children n(%)	CALD children n(%)	aOR	95% Confidence interval		P Value
				Lower	Upper	
Potentially preventable hospitalisations	2,168 (10.63%)	377 (9.12%)	1.28	1.13	1.45	< 0.0001
Hospital re-admission(s)	12,157 (59.6%)	2,759 (66.80%)	1.49	1.37	1.62	< 0.0001
Emergency department presentations without admission(s)	5,918 (29.02%)	1,178 (28.52%)	1.23	1.14	1.34	< 0.0001
Development of chronic health conditions	2,985 (15.63%)	571 (13.829%)	1.44	1.29	1.60	< 0.0001
Stillbirth	1,022 (5.01%)	255 (6.17%)	1.08	0.92	1.26	< 0.0001
Prematurity	4,185 (20.52%)	2,655 (64.27%)	0.55	0.50	0.59	< 0.0001
APGAR < 4	1,635 (8.02%)	406 (9.83%)	1.02	0.90	1.16	< 0.0001

The results from Table 3a show that CALD infants born low birthweight compared to CALD infants born with regular birthweight have higher rates of all health service usage, except GP consultations. Additionally, low birthweight CALD infants had rates of specialist consultations 168.51 times higher than non-low birthweight CALD infants. For infants of Australian-born women, low birthweight infants had higher rates of in-patient episodes, and diagnostic imaging. Additionally, CALD infants born low birthweight are 134.83 times more likely to have hospital outpatient episodes, compared to non-LBW CALD infants.

**Table 3:** Health service use of CALD infants born low birthweight compared to CALD infants born with regular birthweight

(a) Health service	LBW CALD infants	Non-LBW CALD infants		Confidence interval (95%)	
	Mean ± SD	Mean ± SD	% Difference	Lower	Upper
In-patient episode(s)	0.99 ± 2.40	0.57 ± 1.59	75.72	65.42	86.66
Hospital outpatient episode(s)	4.53 ± 11.57	2.02 ± 6.09	134.83	118.87	151.99
ED presentations	4.94 ± 4.04	4.33 ± 3.70	13.96	8.18	20.06
GP consultation(s)	25.58 ± 21.45	27.04 ± 18.52	− 4.82	− 7.14	− 2.43
Specialist consultation(s)	3.86 ± 9.68	1.39 ± 3.46	168.51	152.44	185.59
Pathology tests	4.19 ± 10.78	3.10 ± 6.93	35.60	28.15	41.38
Diagnostic imaging	1.01 ± 2.50	0.69 ± 1.57	46.59	38.09	55.63

(b) LBW	Non-CALD infants	CALD infants		Confidence interval (95%)	
	Mean ± SD	Mean ± SD	% Difference	Lower	Upper
In-patient episode(s)	1.57 ± 3.80	0.99 ± 2.40	− 0.22	− 1.22	0.78
Hospital outpatient episode(s)	6.61 ± 16.88	4.53 ± 11.57	1.01	− 0.19	2.20
ED presentations	6.15 ± 5.96	4.94 ± 4.04	2.35	2.02	2.69
GP consultation(s)	25.01 ± 19.48	25.58 ± 21.45	2.90	2.37	3.43
Specialist consultation(s)	5.00 ± 11.91	3.86 ± 9.68	0.87	− 0.29	1.97
Pathology tests	4.68 ± 15.86	4.19 ± 10.78	0.69	− 0.30	1.68
Diagnostic imaging	1.32 ± 3.46	1.01 ± 2.50	− 0.32	− 1.32	0.68

The results from Table 3b show the health service usage of non-CALD vs. CALD infants that are born with low birthweight. These results show that for all health service usage except for In-patient episodes and diagnostic imaging, non-CALD LBW infants had higher rates of health service usage compared to LBW CALD infants. ED presentations for Non-CALD LBW infants were 2.35 times higher (6.15 ± 5.96 vs. 4.94 ± 4.04), and GP consultations were 2.90 times higher (25.01 ± 19.48 vs. 25.58 ± 21.45).

## Discussion

The aims of this study were 1: to identify the population-level health service utilization and health events and outcomes of the heterogeneous group of CALD infants and children born in Queensland, Australia, who were born with low birthweight between 1 and 2012 to 30th June 2019, in comparison to CALD infants born at regular birthweight, 2: to identify the costs associated with CALD infants accessing health services between birth and 5 years of age for CALD children born with low birthweight compared to CALD children born with birthweight above 2500 g, and 3: to compare low birthweight inequalities and outcomes in early childhood between infants of CALD women and infants of Australian-born women.

Low birthweight CALD infants had higher mean and standard deviation of all health events and outcomes; potentially preventable hospitalisations, hospital re-admissions, ED presentations without admissions, and development of chronic diseases. Additionally, low birthweight CALD infants have higher rates of all health service usage, except GP consultations. Low birthweight CALD infants had rates of specialist consultations 168.51 times higher than non-low birthweight CALD infants.

The results from this study indicate the importance of collecting detailed health service use datain order to understand the health service use of parents and children affected by low birthweight. This would detect inequalities in access to health services and better allow for appropriate planning of health care services and resource allocation for CALD women in Australia. Furthermore, the results from this study highlight the disparities in health events and outcomes and health service access associated with low birthweight infants, between both CALD and Australian born women. The impact of low birthweight on the Australian economy has not been widely recognized. As such, this study has responded to this knowledge gap by identifying that there are significant costs to individuals and governments both directly and indirectly due to low birthweight.

One of the strengths of this study is this paper and its results fill a number of gaps in CALD research, and provide substantial information regarding health service use and health events and outcomes of low birthweight for CALD women in Australia. The use of administrative data is a key strength of this study, as it allows all incidences of low birthweight within a defined population to be identified and included in the economic analyses. Additionally, the use of administrative data reduces the potential for recall bias, that could be introduced if patient surveys were used to identify health service use and cost (Callander et al., 2020). A limitation of this study was the use of data from only one state in Australia, Queensland, to make inferences about the health access and health service useof low birthweight infants across the country. Another limitation is that intrauterine growth restriction (IUGR) data was not used. Another limitation of this study was based on the definition of CALD by the Australian Bureau of Statistics, and the data available, non-English speaking mothers born in Australia were categorised into the non-CALD group. This could have potentially brought the data in this group closer to the mean. It is recommended that further research be conducted with data including language spoken and country of birth to ensure a truly accurate representation of CALD is provided. An additional limitation is that the birthweight categories were not further categorised to including low birthweight and very low birthweight. The authors are aware of the importance of very low birthweight studies and state that conducting a sub analysis of very low birthweight in this paper are beyond the scope of this analyses. An additional limitation is that data for mothers who were born in Australia but do not speak English at home were not included in the analysis, and as this could not be adjusted for, could have impacted the interpretation of the results by the authors. Comparing CALD mothers to Australian-born mothers as two distinct groups was the aim of the project. While it is likely that BMI and smoking are significant mediators, the aim of this study was to provide policy makers with information that differs these two groups—hence we didn’t adjust for BMI and smoking. Further analysis with these additional covariates/confounders/mediators/moderators would identify the contribution arising from them.

One-third of all births in Australia each year are by women who are culturally and linguistically diverse. CALD infants born with low birthweight had higher rates of all health events and outcomes; potentially preventable hospitalisations, hospital re-admissions, ED presentations without admissions, and development of chronic diseases compared to CALD infants born above 2500 g.

## Conclusion

In conclusion, culturally and linguistically diverse women in Australia face a number of barriers to accessing maternity care that Australian born women do not have. Changes should be made to the delivery and access of maternal healthcare in Australia to endeavour that all women receive equal and appropriate care. The barriers to maternal healthcare for CALD women such as cultural differences, limited health literacy, transport issues, and limited financial capacity can also be applicable to other marginalised Australian women. Therefore, an overhaul of Australia’s delivery of maternal care may contribute to a decrease in low birthweight infants, and subsequent health events and outcomes for all marginalised women and infants in Australia.

### Supplementary Information

Below is the link to the electronic supplementary material.

Supplementary material 1 (DOCX 21.2 kb)

## Data Availability

Due to the restrictions of our ethics approval, we are unable to share out data.

## Code Availability

Due to the restrictions of our ethics approval, we are unable to share out data.
