# Supplementary material for: Identifying Longer-Term Health Events and Outcomes and Health Service Use of Low Birthweight CALD Infants in Australia
Source: Matern Child Health J. 2023 Nov 18;28(4):649–56. doi: 10.1007/s10995-023-03819-w (PMC10963444; doi:10.1007/s10995-023-03819-w)
Supplement: Supplementary file 1 — Supplementary material 1 (DOCX 21.2 kb) [file 10995_2023_3819_MOESM1_ESM.docx]

**Supplementary Information**

Table 5: *Characteristics of women who gave birth to LBW and non-LBW CALD infants from 1^st^ July 2012 to 30^th^ June 2018 by CALD sub-group*

| Characteristics | SEA Women | | African Women | | European Women | | Other Asian Women | | Central South American Women | |
| --- | --- | --- | --- | --- | --- | --- | --- | --- | --- | --- |
|  | **LBW** | **Not LBW** | **LBW** | **Not LBW** | **LBW** | **Not LBW** | **LBW** | **Not LBW** | **LBW** | **Not LBW** |
| Mother’s Age at birth |  |  |  |  |  |  |  |  |  |  |
| <20 | 18 (0.13%) | 207 (1.49%) | 22 (0.25%) | 295 (3.4%) | Low Cell Count | 41 (0.59%) | 9 (0.04%) | 82 (0.33%) | Low Cell Count | 20 (0.77%) |
| 21-34 | 624 (4.5%) | 9428 (68.02%) | 392 (4.52%) | 6200 (71.53%) | 222 (3.2%) | 4705 (67.79%) | 1371 (5.45%) | 18941 (75.25%) | 86 (3.31%) | 1612 (61.98%) |
| 35+ | 351 (2.53%) | 3233 (23.32%) | 125 (1.44%) | 1634 (18.85%) | 105 (1.51%) | 1864 (26.85%) | 372 (1.48%) | 4395 (17.46%) | 76 (2.92%) | 805 (30.95%) |
| Mother’s pre-pregnancy BMI, |  |  |  |  |  |  |  |  |  |  |
| Underweight (<18.5) | 157 (1.17%) | 1447 (10.74%) | 72 (0.88%) | 521 (6.35%) | 54 (0.8%) | 467 (6.9%) | 268 (1.09%) | 2596 (10.51%) | 14 (0.56%) | 114 (4.55%) |
| Normal (18.5-24.9) | 570 (4.23%) | 8040 (59.7%) | 236 (2.87%) | 4039 (49.2%) | 202 (2.99%) | 4511 (66.68%) | 1020 (4.13%) | 15048 (60.94%) | 89 (3.55%) | 1571 (62.66%) |
| Pre-obesity (25.0-29.9) | 179 (1.33%) | 2253 (16.73%) | 130 (1.58%) | 2089 (25.44%) | 49 (0.72%) | 1117 (16.51%) | 335 (1.36%) | 4300 (17.41%) | 37 (1.48%) | 508 (20.26%) |
| Obesity class I (30-34.9) | 57 (0.42%) | 699 (5.19%) | 66 (0.8%) | 981 (11.95%) | 17 (0.25%) | 315 (4.66%) | 87 (0.35%) | 961 (3.89%) | 14 (0.56%) | 142 (5.66%) |
| Obesity class II (30-39.9+) | 5 (0.04%) | 61 (0.45%) | 5 (0.06%) | 71 (0.86%) | Low Cell Count | 31 (0.46%) | Low Cell Count | 76 (0.31%) | Low Cell Count | 16 (0.64%) |
| Area-based Socioeconomic Status at birth |  |  |  |  |  |  |  |  |  |  |
| Quintile 1 (Lowest), N (%) | 37 (0.27%) | 458 (3.34%) | 9 (0.1%) | 124 (1.44%) | 10 (0.15%) | 149 (2.17%) | 21 (0.08%) | 374 (1.49%) | Low Cell Count | 22 (0.86%) |
| Quintile 2, N (%) | 18 (0.13%) | 306 (2.23%) | 5 (0.06%) | 99 (1.15%) | 7 (0.1%) | 140 (2.04%) | 39 (0.16%) | 405 (1.61%) | Low Cell Count | 16 (0.62%) |
| Quintile 3, N (%) | 223 (1.62%) | 3031 (22.08%) | 141 (1.63%) | 2085 (24.17%) | 57 (0.83%) | 1151 (16.78%) | 301 (1.2%) | 3817 (15.22%) | 22 (0.86%) | 305 (11.87%) |
| Quintile 4, N (%) | 296 (2.16%) | 3710 (27.03%) | 165 (1.91%) | 2718 (31.51%) | 145 (2.11%) | 2573 (37.51%) | 422 (1.68%) | 5989 (23.88%) | 59 (2.3%) | 878 (34.18%) |
| Quintile 5 (Highest), N (%) | 395 (2.88%) | 5252 (38.26%) | 214 (2.48%) | 3067 (35.55%) | 102 (1.49%) | 2526 (36.82%) | 956 (3.81%) | 12760 (50.87%) | 69 (2.69%) | 1192 (46.4%) |
| Rurality of residence at birth |  |  |  |  |  |  |  |  |  |  |
| Major city, N (%) | 615 (4.48%) | 8150 (59.38%) | 381 (4.42%) | 5720 (66.3%) | 204 (2.97%) | 4374 (63.76%) | 1356 (5.41%) | 18058 (71.99%) | 109 (4.24%) | 1934 (75.28%) |
| Inner Regional, N (%) | 116 (0.85%) | 1525 (11.11%) | 86 (1%) | 1283 (14.87%) | 45 (0.66%) | 944 (13.76%) | 140 (0.56%) | 1886 (7.52%) | 25 (0.97%) | 227 (8.84%) |
| Outer Regional, N (%) | 168 (1.22%) | 2297 (16.73%) | 46 (0.53%) | 751 (8.71%) | 59 (0.86%) | 883 (12.87%) | 185 (0.74%) | 2658 (10.6%) | 16 (0.62%) | 203 (7.9%) |
| Remote and Very Remote, N (%) | 70 (0.51%) | 785 (5.72%) | 21 (0.24%) | 339 (3.93%) | 13 (0.19%) | 338 (4.93%) | 58 (0.23%) | 743 (2.96%) | 6 (0.23%) | 49 (1.19%) |
| Mother’s Tobacco smoking status at 20 weeks’ gestation |  |  |  |  |  |  |  |  |  |  |
| Yes, N (%) | 32 (0.23%) | 345 (2.49%) | 25 (0.29%) | 164 (1.89%) | 32 (0.46%) | 289 (4.16%) | 9 (0.04%) | 141 (0.56%) | Low Cell Count | 39 (1.5%) |
| No, N (%) | 954 (6.88%) | 12484 (90.07%) | 509 (5.87%) | 7948 (91.69%) | 291 (4.19%) | 6307 (90.87%) | 1728 (6.87%) | 23254 (92.93%) | 157 (6.04%) | 2392 (91.96%) |
| Marital Status |  |  |  |  |  |  |  |  |  |  |
| Divorced | 10 (0.07%) | 111 (0.8%) | 5 (0.06%) | 53 (0.61%) | Low Cell Count | 31 (0.45%) | 8 (0.03%) | 50 (0.2%) | Low Cell Count | 16 (0.62%) |
| Married (Registered) | 141 (1.02%) | 1963 (14.16%) | 80 (0.92%) | 1194 (13.77%) | 47 (0.68%) | 1074 (15.47%) | 335 (1.33%) | 4201 (16.69%) | 30 (1.15%) | 445 (17.11%) |
| Married/ De Facto | 716 (5.17%) | 9650 (69.62%) | 343 (3.96%) | 5692 (65.67%) | 236 (3.4%) | 5075 (73.12%) | 1348 (5.36%) | 18391 (73.07%) | 116 (4.46%) | 1840 (70.74%) |
| Never Married | 106 (0.76%) | 977 (7.05%) | 88 (1.02%) | 998 (11.51%) | 33 (0.48%) | 348 (5.01%) | 55 (0.22%) | 712 (2.83%) | 18 (0.69%) | 123 (4.73%) |
| Not Stated/ Unknown | Low Cell Count | 6 (0.04%) | Low Cell Count | Low Cell Count | 8 (0.12%) | 52 (0.75%) | Low Cell Count | 13 (0.05%) | Low Cell Count | Low Cell Count |
| Separated | 16 (0.12%) | 147 (1.06%) | 19 (0.22%) | 168 (1.94%) | Low Cell Count | 29 (0.42%) | 6 (0.02%) | 48 (0.19%) | Low Cell Count | 11 (0.42%) |
| Widowed | Low Cell Count | 14 (0.1%) | Low Cell Count | 23 (0.27%) | Low Cell Count | Low Cell Count | Low Cell Count | Low Cell Count | Low Cell Count | Low Cell Count |
| PREV Preg |  |  |  |  |  |  |  |  |  |  |
| Yes | 620 (4.47%) | 8872 (64.01%) | 385 (4.44$) | 6087 (70.22%) | 174 (2.51%) | 4223 (60.84%) | 923 (3.67%) | 14085 (55.96%) | 88 (3.38%) | 1424 (54.75%) |
| No | 373 (2.69%) | 3996 (28.83%) | 154 (1.78%) | 2042 (23.56%) | 157 (2.26%) | 2387 (34.39%) | 829 (3.29%) | 9333 (37.08%) | 76 (2.92%) | 1013 (38.95%) |
| Gest 1^st^ ANC visit |  |  |  |  |  |  |  |  |  |  |
| <20 weeks | 869 (6.27%) | 11252 (81.18%) | 471 (5.43%) | 7119 (82.13%) | 298 (4.29%) | 6090 (87.74%) | 1601 (3.67%) | 21403 (85.03%) | 152 (5.84%) | 2279 (87.62%) |
| 20+ weeks | 124 (0.89%) | 1616 (11.66%) | 68 (0.78%) | 1010 (11.65% | 33 (0.48%) | 520 (7.49%) | 151 (0.6%) | 2015 (8.01%) | 12 (0.46%) | 158 (6.07%) |

Table 6: *Health service use of CALD children born LBW compared to CALD children not born LBW from birth to age 5*

| Health Service | LBW CALD children mean (SD) | | | | | Non-LBW CALD children mean (SD) | | | | |
| --- | --- | --- | --- | --- | --- | --- | --- | --- | --- | --- |
|  | Child age (months) | | | | | Child age (months) | | | | |
|  | **0-12** | **13-24** | **25-36** | **37-48** | **49-60** | **0-12** | **13-24** | **25-36** | **37-48** | **49-60** |
| In-Patient Episode(s) | 0.50±1.35 | 0.22±0.78 | 0.14±0.63 | 0.09±0.47 | 0.05±0.36 | 0.22±0.66 | 0.15±0.61 | 0.09±0.52 | 0.07±0.52 | 0.04±0.41 |
| Hospital Outpatient Episode(s) | 7.34±9.75 | 5.02±7.81 | 4.30±6.85 | 3.64±4.82 | 3.93±5.04 | 4.27±5.29 | 3.02±5.05 | 3.14±5.41 | 3.11±5.33 | 3.23±5.38 |
| ED Presentations | 0.69±1.31 | 0.57±1.18 | 1.74±1.27 | 0.20±0.67 | 0.12±0.52 | 0.60±1.17 | 0.54±1.07 | 1.62±1.11 | 0.21±0.65 | 0.12±0.49 |
| GP Consultation(s) | 7.97±6.60 | 6.82±6.49 | 4.52±5.30 | 3.67±4.75 | 2.59±3.80 | 8.80±6.11 | 7.21±5.97 | 4.61±4.72 | 3.75±4.24 | 2.68±3.57 |
| Specialist Consultation(s) | 2.58±7.89 | 0.52±1.47 | 0.33±1.10 | 0.26±0.93 | 0.17±0.68 | 0.62±1.71 | 0.25±1.03 | 0.20±0.94 | 0.18±0.82 | 0.14±0.73 |
| Pathology Tests | 1.76±9.39 | 0.87±2.09 | 0.65±1.76 | 0.55±1.65 | 0.36±1.20 | 0.78±3.36 | 0.77±2.27 | 0.61±2.12 | 0.54±1.95 | 0.41±1.64 |
| Diagnostic Imaging | 0.48±1.86 | 0.18±0.63 | 0.14±0.51 | 0.11±0.45 | 0.10±0.45 | 0.23±0.79 | 0.15±0.57 | 0.12±0.53 | 0.11±0.48 | 0.08±0.40 |
